# Potential Impact of HLA DQB1*05 on Identical Sibling Hematopoietic Stem Cell Transplantation Outcome †

**DOI:** 10.3390/jcm14196798

**Published:** 2025-09-26

**Authors:** Fatma Al Lawati, Murtadha Al Khabori, Salma Al Harrasi, Aliya Al Ansari

**Affiliations:** 1 College of Science, Sultan Qaboos University, Muscat 123, Oman; 2 National Genetic Center, Ministry of Health, Muscat 100, Oman; 3 College of Medicine and Health Sciences, Sultan Qaboos University, Muscat 123, Oman; khabori@squ.edu.om; 4 National Hematology and Bone Marrow Transplant Center, University Medical City, Muscat 123, Oman

**Keywords:** hematopoietic stem cell transplantation, HLA-identical siblings, graft versus host disease, HLA-DQB1*05

## Abstract

**Background/Objectives:** Human leukocyte antigens (HLAs) are major determinants of successful allogeneic hematopoietic stem cell transplantation (allo-HSCT). Their alleles are closely linked to outcomes, even in HLA-identical sibling donor (ISD) HSCT. This retrospective study analyzed the impact of HLA alleles on HLA-ISD HSCT outcomes in Omani patients. **Methods:** Data were collected for a heterogenous cohort of patients registered at the Sultan Qaboos University Hospital (SQUH), who underwent HLA-ISD HSCT from 2012 to 2022 (*n* = 153). HSCT outcomes, namely acute GVHD (aGVHD), chronic GVHD (cGVHD), chimerism status (complete or mixed) at 6 to 12 months after HSCT, neutrophil and platelet engraftment time, and patient five-year overall survival, were included. Low-resolution HLA-typing records were collected for five HLA loci: HLA-A, B, C, DRB1 and DQB1. GVHD and chimerism were analyzed by logistic regression analysis. Platelet and neutrophil engraftment times were assessed by Mann–Whitney tests. Patient overall survival was evaluated by the Kaplan–Meier model and Log-rank testing. At a 95% confidence interval, the *p*-value threshold was corrected using Bonferroni correction. **Results:** The incidence rates of aGVHD and cGVHD from all grades were 16% and 15%, respectively. Although no association between HLA alleles or any of the investigated outcomes was identified, survival curve analyses indicated a significant protective effect of HLA-DQB1*05 (*p* = 0.01). Patients carrying this allele had a better-estimated 5-year overall survival (90%) than did DQB1*05 negative patients (68%). **Conclusions:** This study suggests that HLA-DQB1*05 in the Omani population could have an impact on overall survival and might be a predictive biomarker. Further studies on a larger scale in other regional populations are needed to validate our findings and explore the underlying mechanism.

## 1. Introduction

Allogenic hematopoietic stem cell transplantation (allo-HSCT) is a curative therapy for several malignant and nonmalignant hematological disorders. The matching status of human leukocyte antigens (HLAs) between donors and recipients is a major factor for a successful transplantation. Mismatches in classical HLA genes (HLA-A, B, C, DRB1, DQB1, and DPB1) are associated with adverse HSCT outcomes such as non-engraftment, acute or chronic graft versus host disease (aGVHD and cGVHD, respectively), reduced overall survival, and reduced disease-free survival after HSCT [1]. Therefore, an HLA-identical sibling donor (ISD) transplant is the best choice of donor if available, as it is associated with reduced risk of GVHD, increased overall survival (OS), and enhanced engraftment [2]. Nevertheless, adverse HSCT outcomes could also occur in HLA-ISD allo-HSCT that were attributed to genetic contributors within or outside the HLA genomic cluster [3].

Several studies reported significant associations between HSCT outcomes and HLAs in HLA-ISD HSCT. Two studies from Switzerland and the UK showed that HLA-DR*15 is significantly associated with reduced relapse rate and improved survival in patients with leukemia or non-Hodgkin lymphoma after HLA-ISD HSCT [4,5]. Similarly, a large study (*n* = 1204) reported a significant impact of HLA DR15 in reducing secondary graft failure after HLA-ISD HSCT [6]. An Iranian study found several HLA associations. HLA-B*07 was linked to a higher incidence of acute graft-versus-host disease (aGVHD) (grades II-IV), while HLA-A*01, B*40, B*41, and B*57 significantly increased the risk of chronic GVHD (cGVHD). Regarding mortality, HLA-DRB1*02, B*13, B*40, and DRB1*04 were significantly associated with an increased death rate. HLA-DRB1*15 showed a non-significant trend towards a decreased incidence of aGVHD [7]. In a Slovakian study, recipients carrying the HLA-A*01, -DRB1*03 and -DQB1*03 alleles showed lower incidence of aGVHD, while the HLA-DQB1*06 allele was associated with a higher incidence of cGVHD [8]. Another study on Japanese patients with leukemia or myelodysplastic reported an association of HLA-B*60 with an increased risk of aGVHD at grades II-IV, and HLA B*62 with decreased grades II-IV [3]. A recent report from a dataset of Japanese patients with adult T-cell leukemia/lymphoma (ATL) revealed that HLA-B60 was associated with an increased risk of acute GVHD and overall mortality, while HLA-B62 was associated with a decreased risk of overall and transplant related mortality [9]. Based on existing research, it is evident that replication of findings may be observed only when studies are conducted within the same ethnic group. This may indicate that the HLA contribution to unfavorable HSCT outcomes may differ globally due to variations in HLA genomics, specifically linkage disequilibrium blocks, and the unique distribution of HLA alleles across distinct ethnicities.

Currently, there is a gap in research from Arab countries regarding the impact of HLA alleles on HSCT outcomes. Our study aims to address this by investigating an Omani cohort, marking the first such report from an Arab population. Allogenic HSCT was conducted in Oman in a single center at Sultan Qaboos University Hospital (SQUH), with 84% of procedures utilizing HLA-ISDs and a reported 18% risk of aGVHD (Grades II-IV) and an 8% risk of cGVHD following transplants [10]. The current study hypothesized that individual HLAs might affect the clinical outcomes of patients after HLA-ISD HSCT and therefore retrospectively evaluated the influence of HLA alleles on several HSCT outcomes. The study could refine risk assessment and outcome prediction for HSCT recipients and improve post-HSCT management.

## 2. Materials and Methods

### 2.1. Patients

Data were collected retrospectively for 153 registered patients, who underwent allo-HSCT from fully HLA-matched related donors in the period from 2012 to 2022. All donors were siblings except one, who was an HLA identical mother. The baseline parameters collected were the date of HSCT, donor and recipient demographics (age, gender, blood group), CMV status, stem cell characteristics (source and dose), conditioning regimen, and GVHD prophylaxis.

### 2.2. Inclusion and Exclusion Criteria

The study included both male and female patients who received matched sibling transplants, either at SQUH or elsewhere if their early post-transplant care was managed at SQUH. The inclusion period covered January 2012 to December 2022. Patients with HLA reports missing, incomplete clinical data or with less than seven days survival after HSCT were excluded. The characteristics of patients are shown in Table 1.

### 2.3. Outcomes

We assessed several HSCT outcomes, including aGVHD, an inflammatory condition occurring within 100 days of transplantation, and cGVHD, which develops after 100 days and exhibits autoimmune-like characteristics [11,12]. We also analyzed patient chimerism status (complete or mixed) at 6 to 12 months post-HSCT. Chimerism refers to the proportion of donor and recipient cells in the recipient’s blood or bone marrow after HSCT. Complete chimerism indicates only donor cells are present, while mixed chimerism shows both donor and recipient cells, with 5–95% of cells being of donor origin [13].

Moreover, the study examined clinical parameters including neutrophil and platelet engraftment times following HSCT, and five-year overall patient survival. Neutrophil engraftment was defined as a sustained neutrophil count exceeding 500 × 10^6^/L over three consecutive days [14], while platelet engraftment required seven days of transfusion independence, and a platelet count above 20 × 10^9^/L [15].

Data were retrieved from the HLA laboratory at Sultan Qaboos University (SQU). For samples collected between 2012 and 2014, serological techniques were used for HLA class I typing of HLA-A, B and C while PCR-SSP was employed for HLA class II genotyping (HLA-DRB1 and DQB1). From 2012 to 2014, the available genotypes were at a low-resolution level for HLA class I (serological methods) and HLA class (PCR-SSP technique) for patients and donors. We converted class I serotypes to the most probable molecular HLA alleles according to the WHO-HLA Nomenclature Committee [16]. Genotypes after 2014 were typed as class I and II on the Luminex platform (Luminex Corp., Austin, TX, USA), which is based on the sequence-specific oligonucleotide (SSO) method. For all patients, five HLA loci, HLA-A, B, C, DRB1 and DQB1, were reported at a low-resolution level (two digits).

## 3. Results

### 3.1. Baseline Characteristics

This study involved 153 patients, categorized as having either malignant (62 patients) or non-malignant (91 patients) hematological disorders. Most of the patients were males (61%) and pediatric cases (below 18 years old) comprised 37.9% of the cohort (Table 1).

At the time of transplant, the patients’ median age was 21 years. Peripheral blood was the primary source of stem cells (68.6%). Over half the patients (55.8%) received fludarabine, busulfan, and anti-thymocyte globulin (Flu/Bu/ATG) for conditioning, and most (64.7%) were given cyclosporine (CSA) for GVHD prevention. Neutrophil and platelet engraftment occurred at a median of 14 and 15 days, respectively. The incidence of acute and chronic GVHD (all grades) was 16% and 15%, respectively. Within 6 to 12 months post-HSCT, mixed chimerism was observed in 20.3% of patients, while 2% experienced complete rejection.

### 3.2. GVHD, Engraftment and Chimerism

**Figure 1:**
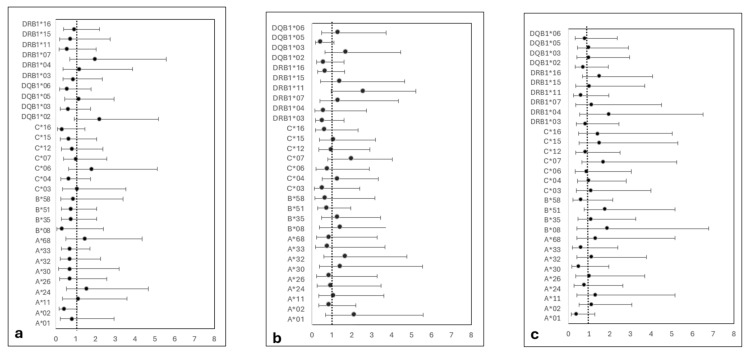
HLA allele risk ratios for (**a**) aGVHD, (**b**) cGVHD, and (**c**) mixed chimerism, displayed in a forest plot. The dashed vertical line at 1.00 indicates the risk ratio. Error bars represent the confidence interval at 95%. All *Pc*-values are >0.05.

Univariate regression analysis revealed no significant association between the selected HLAs and the risk of aGVHD, cGVHD, chimerism status, or engraftment (Figure 1). However, the HLA DQB1*05 allele was associated with a trend of decreased cGVHD risk but did not reach significance (OR 0.43, CI (0.17–1.09) and *p* = 0.07). Detailed ratios and *p*-values are available in Supplementary Table S1.

**Figure 2:**
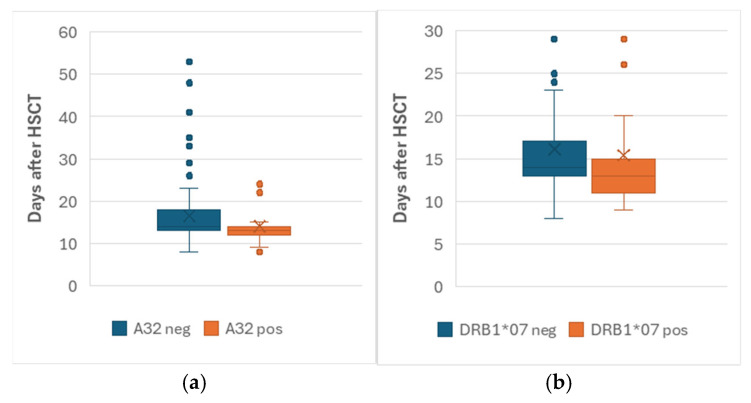
Box plots of neutrophil engraftment time among: (**a**) HLA A*32 negative and positive patients; (**b**) HLA-DRB1*07 negative and positive patients. Median values are represented by horizontal lines inside the boxes. The dots are data outliers (patients with long engraftment time). The crosses are average engraftment times.

Also, there was no significant association between HLA and neutrophil or platelet engraftment times (Table S2). Although trends of earlier neutrophil engraftment in HLA A*32 and DRB1*07 carriers (medians 13 vs. 14 days) were observed (Figure 2), these did not achieve statistical significance after correction (*Pc* = 0.23 and 0.18, respectively).

### 3.3. Overall Survival

**Figure 3:**
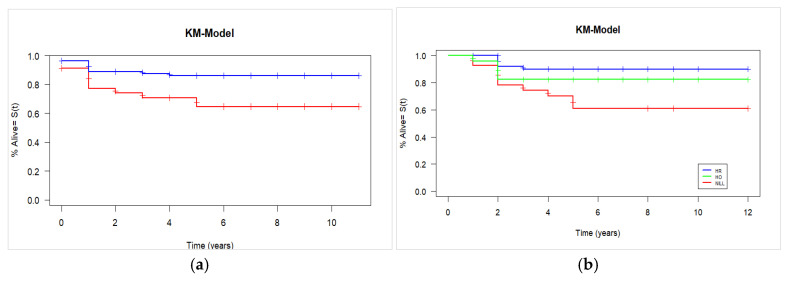
Kaplan–Meier probability of overall survival plots for (**a**) DQB1*05- carriers (blue line) and DQB1*05-non carriers (red line). (**b**) DQB1*05 genotype. The blue, green and red lines indicate heterozygotes, homozygotes and no-carriers, respectively.

With a median follow-up of five years, 126 patients (82.4%) were alive, and statistically significant better survivals were associated with the presence of HLA-DQB1*05 (*p* = 0.010, *Pc* = 0.04). The presence of DQB1*05 correlated with improvement in estimated 5-year overall survival, with rates of 90% (95% CI: 88–93%) compared to 68% (95% CI: 65–73%) in its absence, as illustrated in Figure 3a. Using the Cox proportional hazard model, the hazard ratio for carrying DQB1*05 was 0.39 (95% CI, 0.18 to 0.84, *p*-value = 0.01, *Pc* = 0.05). The Cox model revealed also that age, gender and source of stem cell were not statistically significant predictors of the overall survival (*p*-value = 0.52, 0.20 and 0.69, respectively).

We analyzed the effect of DQB1*05 genotype (heterozygous, homozygous, or non-carrier) on overall survival. The log-rank test revealed a significant difference (*p* = 0.01), driven by a significant decrease in survival among non-carriers compared to both heterozygous and homozygous carriers. However, no significant difference in survival was observed between DQB1*05 heterozygous and homozygous patients (Figure 3b).

We found also a trend of better survival in HLA-DRB1*16 carriers, but it did not remain significant after multiple testing correction (*p*-value = 0.05, *Pc* = 0.3). No significant differences in survival curves were observed between HLA-positive and HLA-negative patients for the remaining HLA alleles (Table S3).

## 4. Discussion

The current study is the first to analyze HLA-ISD HSCT outcomes in Oman and, uniquely, explores the influence of HLA on HSCT outcomes. Despite the small cohort size, our patient’s recovery pattern agreed almost with the published reported ranges and our population had lower incidences of both aGVHD and cGVHD (Table 2).

Given the heterogeneity of the study population, we also compared HSCT outcomes within the cohort subgroups based on age, disease type, sex match and source of stem cell Table S4. The values in this cohort were comparable to the existing literature, with very low cGVHD incidence in children (7.40%) and in bone marrow transplant patients (4.65%).

**Figure 4:**
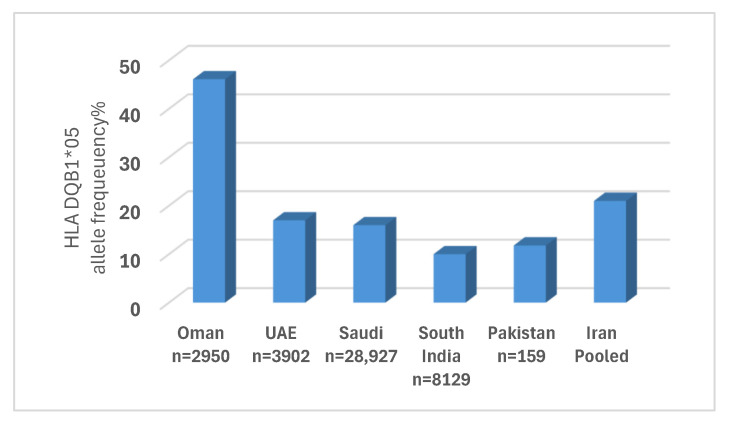
HLA DQB1*05 allele frequency percentage in Oman and neighboring countries.

Among 30 HLA alleles assessed, HLA-DQB1*05 was the only significant predictor (*Pc* = 0.048), associated with improved 5-year OS. The HLA-DQB1*05 allele is very common in Oman (46.1% frequency) [27], and we found a similar frequency (48.7%) in our HSCT cohort. Its high prevalence has likely enhanced the detection of this association, even with a small sample size [28]. Despite the high prevalence (over 10%) of this allele in neighboring countries like the United Arab Emirates [29], Saudi Arabia [30], South India [31], Pakistan [32], and Iran [33], Oman has an exceptionally high frequency reaching nearly half of the population (Figure 4).

We postulated that the protective effect of HLA-DQB1*05 allele might be mediated through lowering cGVHD incidence, as DQB1*05 exhibited the lowest odds ratio (RR: 0.87, *p* = 0.07, Figure 1b). Notably, cGVHD has an autoimmune-like underlying pattern [20] and DQB1*05 and its alleles were shown to confer protection against autoimmune diseases like type 1 diabetes in Arab populations [21,34]. Thus, DQB1*05 alleles may modulate post-HSCT immune responses and decrease cGVHD incidence.

Interestingly, the HLA-DQB1*05 allele behaved differently in children and adults. For children, its frequency was nearly identical in those who developed chronic graft-versus-host disease (75%) and those who did not (74%). For adults, however, the allele was much more common in patients who did not develop cGVHD (74%) compared to those who developed it (50%). This disparity suggests a potential protective role of the allele in adult patients.

The underlaying mechanisms for HLA association with HSCT outcomes remain to be elucidated. Clearly, different outcomes, such as aGVHD and cGVHD are associated with different immunological patterns and thus different HLA Class I and or II alleles. Also, potentially the association might not be only due to antigen presentation, as some studies have associated differential HLA allele-specific expression with HSCT outcomes. For example, aGVHD incidence was greater in patients with highly expressed HLA-DPB1 alleles who received an HLA-DPB1 mismatched transplant from a donor with low expression HLA-DPB1 alleles [35].

The main finding of this study was the association between HLA-DQB1*05 and improved five-year overall survival in Omani patients undergoing HLA-ISD HSCT. This observation highlights that allo-HSCT outcomes may be influenced not only by HLA allele matching but also by the specific HLA allele itself and the patient’s genetic background. The study is opening the path for further researchers to prove or disprove their observation about a specific HLA allele that may have a role in a better outcome of such stem cell operations.

It is worth mentioning here that HLA A*32 and DRB1*07 carriers showed a trend of faster neutrophil engraftment that became insignificant after correction. This weak association might be attributed to their lower frequencies in the population, and the relatively small cohort size. Larger studies are needed to confirm this observation.

As the first retrospective study to evaluate the association of HLA alleles with HSCT outcomes, several limitations were identified that should be addressed in future research. First, a small sample size potentially influenced the identification of association, especially with relatively uncommon alleles. Second, a heterogeneous cohort of adult and pediatric patients with various diseases that may differentially influence HSCT outcome and HLA effect on malignant conditions is not comparable to a cohort of patients with heritable and hyperinflammatory disorders. Third, the use of different approaches (serological and molecular) in HLA class I typing means that the data from the two time periods are not fully comparable and any conclusions drawn from these groups should be interpreted with caution. However, we believe this is a minor limitation because our study’s primary conclusions are drawn from HLA Class II alleles, which were consistently typed with molecular methods from the outset.

Currently, most transplants are from HLA identical siblings or related donors that do not demand high-resolution typing. However, low-resolution typing groups multiple alleles that can differ significantly at the amino acid level, while four-digit typing distinguishes between alleles that may have distinct peptide-binding profiles and immune responses. Moreover, new approaches such as RNA-based next generation sequencing (NGS) enable us to quantify HLA allele-specific expression differences between individuals with different phenotypes.

## 5. Conclusions

In conclusion, from the dataset of a heterogenous cohort of Omani patients who received HLA-ISD HSCT, we have detected a decreased incidence of cGVHD and a significant association with five-year OS with HLA DQB1*05. Consistently, this allele has been reported to be protective in some other autoimmune disorders, suggesting its low immunogenicity. Due to the limited sample size in this study, our findings need to be verified in larger studies. Further research on HLA immunogenicity could be of predictive value for long-term HSCT outcomes and contribute to better post-HSCT management.

## Figures and Tables

**Table 1:** The characteristics of the study patients (*n* = 153).

Patient Variable	*n*	%	Patient Variable	*n*	%
Patient gender			Patient/Donor sex match		
Male	93	61.0	Yes	69	45.0
Female	60	39.0	No	76	49.7
			Unknown	8	5.2
Age group			GVHD prophylaxis		
<18	58	37.9	Cyclosporine-based	99	64.7
18–60	95	62.1	Tacrolimus-based	38	24.8
			CSA and Tacro	15	9.8
Patient/Donor ABO compatibility			Not found	1	1.3
Compatible	119	77.8	Clinical Diagnosis		
Incompatible (major)	19	12.4	AML	26	17.0
Incompatible (minor)	6	3.9	ALL	17	11.1
Incompatible (bidirectional)	2	1.3	MDS	8	8.4
Not recorded	7	4.6	Aplastic anemia	13	5.2
Patient CMV Status			Thalassemia	8	5.2
Pos	129	84.3	Sickle cell disease	39	25.5
Neg	5	3.3	CGD	7	4.6
Not reported	19	12.4	Others (malignant/nonmalignant)	35 (12/23)	22.8
Stem Cell Source					
Bone marrow	43	28.1	Status Jun 2023		
Peripheral blood	105	68.6	Alive/Censored	126	81.8
Both	5	3.2	Dead	27	18.2

AML: acute myeloid leukemia, ALL: acute lymphoblastic leukemia, MDSs: myelodysplastic syndromes; CGDs: chronic granulomatous diseases.

**Table 2:** HLA-ISD HSCT outcomes in the current study compared to published data.

No	Parameter	Study Cohort	Published Data	References
1	Median neutrophil engraftment time	14 days	12–16 days	[17,18,19]
2	Median platelet engraftment time	15 days	14–17 days	[17,18,19]
3	Complete chimerism rate	84.1% (malignant) 68.4% (nonmalignant)	80–90% (malignant) 50–60% (nonmalignant)	[20,21]
4	Acute GVHD rate	16%	20–35%	[22]
5	Chronic GVHD rate	15%	20–50%	[23]
6	5-year overall survival	73% malignant 88.9% nonmalignant	56–75% (malignant) 80–90% (nonmalignant)	[24,25,26]

## Data Availability

No new data were created or analyzed in this study.

## Supplementary Materials

The following supporting information can be downloaded at https://www.mdpi.com/article/10.3390/jcm14196798/s1: Table S1: HLA allele frequency and effect on acute GVHD, chronic GVHD and complete chimerism; Table S2: Impact of HLA alleles on platelet and neutrophil recovery time after HSCT; Table S3: Log rank test results for differences of survival curves in the presence and absence of the study HLA alleles; Table S4: HSCT outcome comparison between the study cohort subgroups.

## Abbreviations

The following abbreviations are used in this manuscript:

Allo-HSCT	Allogenic Hematopoietic Stem Cell Transplantation
ISD	Identical Sibling Donor
HLA	Human Leukocyte Antigen
SQUH	Sultan Qaboos University Hospital
GVHD	Graft versus Host disease
cGVHD	Chronic Graft versus Host Disease
aGVHD	Acute Graft versus Host Disease
OS	Overall Survival
ATL	Adult T cell Leukemia/Lymphoma
CMV	Cytomegalo Virus
WHO	World Health Organisation
SSO	Single Strand Oligonucleotide
CSA	Cyclosporin A
OR	Odd Ratio
